# Knowledge to Predict Pathogens: *Legionella pneumophila* Lifecycle Systematic Review Part II Growth within and Egress from a Host Cell

**DOI:** 10.3390/microorganisms10010141

**Published:** 2022-01-11

**Authors:** Alexis L. Mraz, Mark H. Weir

**Affiliations:** 1 School of Nursing, Health, Exercise Science, The College of New Jersey, P.O. Box 7718, 2000 Pennington Rd., Ewing, NJ 08628, USA; 2 Division of Environmental Health Sciences, College of Public Health, The Ohio State University, Columbus, OH 43210, USA; weirmarkh@gmail.com; 3 Sustainability Institute, The Ohio State University, Columbus, OH 43210, USA

**Keywords:** *Legionella pneumophila*, protozoan host cell, biofilm, premise plumbing, intracellular growth, egress

## Abstract

*Legionella pneumophila* (*L. pneumophila*) is a pathogenic bacterium of increasing concern, due to its ability to cause a severe pneumonia, Legionnaires’ Disease (LD), and the challenges in controlling the bacteria within premise plumbing systems. *L. pneumophila* can thrive within the biofilm of premise plumbing systems, utilizing protozoan hosts for protection from environmental stressors and to increase its growth rate, which increases the bacteria’s infectivity to human host cells. Typical disinfectant techniques have proven to be inadequate in controlling *L. pneumophila* in the premise plumbing system, exposing users to LD risks. As the bacteria have limited infectivity to human macrophages without replicating within a host protozoan cell, the replication within, and egress from, a protozoan host cell is an integral part of the bacteria’s lifecycle. While there is a great deal of information regarding how *L. pneumophila* interacts with protozoa, the ability to use this data in a model to attempt to predict a concentration of *L. pneumophila* in a water system is not known. This systematic review summarizes the information in the literature regarding *L. pneumophila*’s growth within and egress from the host cell, summarizes the genes which affect these processes, and calculates how oxidative stress can downregulate those genes.

## 1. Introduction

### 1.1. Gap in the Literature

Due to the prevalence of *L. pneumophila* in the water system and the seriousness of legionellosis, a set of two respiratory diseases: Legionnaire’s Disease (LD) and the less severe Pontiac Fever, there has been a focus on *L. pneumophila* in the literature. Review articles separately discuss health effects, pathology, treatment, transmission, etiology, epidemiology, and infectivity [1,2,3,4,5,6,7,8,9]. A review article that combines these knowledge goals within the context of modeling the lifecycle of *L. pneumophila* in the water system to forecast concentration is needed. As environmental stress affects the regulation and processing of bacterial genes, it is an integral part in modeling the lifecycle of a pathogen. Data and knowledge pertinent to the impact of environmental stress on a pathogen’s lifecycle must be reviewed to assess their impact on the postulated model framework and the accuracy of a predictive model. In this, a convergence of the above-referenced fields must be reviewed and assessed for use within such a model. A review of this nature does not, to date, exist in the literature.

### 1.2. Need for This Review

This review seeks to summarize the information pertinent to developing a predictive growth model which incorporates the host–cell interactions of *L. pneumophila* and how environmental stressors affect these interactions. The lifecycle of *L. pneumophila*, as it pertains to the host cells involves three processes: uptake, growth, and egression. Considering the complexity of these processes, we have divided this systematic literature review into two parts, the invasion of the host cell [10], and the growth within and egress from the host cell, presented here. This paper focuses on the pathways, processes, and data for forecasting growth and egress of *L. pneumophila* within and from the host cell. Understanding how *L. pneumophila* interacts with host cells in its environment, particularly in biofilms, is vital in understanding how to treat *L. pneumophila* in the water system. Thus far, attempts to eradicate the bacteria from the water system have been made in vain [11,12]. However, understanding the genetics in which *L. pneumophila* can utilize host cells in its environment for protection and increased pathogenesis, and how certain environmental stressors can affect those genes is vital in understanding how to control *L. pneumophila* in the water system.

The mechanisms between *L. pneumophila*’s growth and egress in a protozoa and a human macrophage are similar, and the genetic knockdowns which prevent the bacteria from growing or egressing in a protozoan typically prevent growth and egress within a macrophage as well [13]. Additionally, if *L. pneumophila* is unable to replicate within and egress from a host cell in the environment, the bacteria will be less, or even potentially not at all, infectious to human macrophages [14]. Determining if chlorination or other environmental stressors of the water treatment process influences or causes, genetic knockdowns, which will decrease *L. pneumophila*’s ability to survive and thrive, is a first step in controlling legionellosis outbreaks. This review summarizes knowledge and data needed for a clearer picture of how environmental quality can govern growth within and egress from a host cell. A new means of controlling infectivity may rely on reduced growth or egress from host cells. These data are a vital initial foray into modeling these processes. This study provides said data from the literature which can be used in a predictive model.

### 1.3. General Growth Requirements

*L. pneumophila* thrives in fresh, warm water environments, with supportive nutrients and protozoan hosts [15]. Their ideal temperature range is 20–45 °C, with the bacteria being dormant below 20 °C and typically unable to survive at temperatures higher than 60 °C [16]. *Legionella* has been shown to survive 70 °C treatments for up to 30 min [17]. In premise plumbing systems, *L. pneumophila* utilizes the biofilm for protection against disinfectants and for access to nutrients and host cells, typically amoeba. Biofilms have been shown to be protective against environmental stressors, such as chlorine, to the bacteria [18,19]. The organic matter of the biofilm increases chlorine demand, which maximizes on the surface of the biofilm [20].

The rate in which chlorine penetrates the biofilm is dependent on the density of the biofilm, with older, more established biofilms being denser and therefore more resistant to chlorine [21]. In dense biofilms, as little as 10% of the bulk chlorine will penetrate the biofilm [21]. Growth of *L. pneumophila* in the biofilm is supported by high nutrient levels of iron, organic carbon, nitrogen, and phosphorous [22]. *L. pneumophila* has multiple iron acquisition pathways and is unable to grow in the absence of iron [23,24]. The bacteria produce siderophores to aid in iron acquisition in low iron environments, but are more robust in the high iron environment typical of a biofilm [25,26].

### 1.4. Antibiotic Resistance in L. pneumophila

Antibiotic resistance is of concern for any pathogen, particularly those associated with hospital acquired infections (HAIs). As such, the susceptibility of *L. pneumophila* to antibiotics has been studied since shortly after the discovery of the pathogen [27,28]. While there has been an increase in antibiotic resistance of *L. pneumophila* in environmental samples and patient cultures, there has yet to be a challenge in treating LD infections [29,30]. Research indicates that some antibiotic resistance may be due to environmental conditions and the relationship of the pathogen with host cells [31,32]. There is always the concern that antibiotic strains of *L. pneumophila* will become more virulent and more common place, as seen in the methicillin-resistant *Staphylococcus aureus* (MRSA) [33]. For that reason, it may be prudent to test for antibiotic resistance of *L. pneumophila* in premise plumbing samples, particularly in high risk buildings, such as hospitals [34].

### 1.5. Relationship with Host Cells

*L. pneumophila* is a facultative intracellular bacterium which can utilize host cells, such as amoeba, within the biofilm for further protection and to serve essentially as a unicellular incubator [35]. *L. pneumophila* grown intracellularly are smaller, more highly mobile, and have increased levels of microbial resistance when compared to bacterial progeny not grown in a host cell [31]. *Acanthamoeba polyphaga* (*A. polyphaga*) cysts are protective to *L. pneumophila* with chlorine concentrations as high as 50 mg/L, whereas free-living *L. pneumophila* is susceptible to chlorine concentrations as low as 2 mg/L [36,37].

### 1.6. Implications of This Study

The bacteria can replicate freely within in the host cell and can do so in multiple vacuoles and in counts typically over two dozen bacterium per vacuole [38]. Vacuoles can be expelled from the host cell prior to cell death [39]. The bacteria have been shown to be more infective to mammalian macrophages after having replicated within an amoeba host [35]. It is even theorized that the bacteria will not cause disease in humans unless it has replicated within a protozoan host first [40,41]. Therefore, bacteria growing and egressing from a host cell is a crucial part of *L. pneumophila*’s lifecycle in concern to human infectivity. However, current growth models for *L. pneumophila* in premise plumbing system do not incorporate the interactions with the host cell and the effects of disinfectants on the genes that dictate those interactions. This review is pertinent in summarizing the information needed to build said models.

## 2. Materials and Methods

### 2.1. Search Strategy

To determine the mechanisms, proteins, and genes involved in the growth of *Legionella* within and egress from a host cell, an exhaustive literature review was conducted from inception to January 2019. Google Scholar, PubMed, Scopus, Web of Science, Bioline International, and PLOS ONE were searched using the terms: “((*Legionella*) OR (*Legionella pneumophila*) OR (*L. pneumophila*) OR (*Legionella longbeachae*) OR (*L. longbeachae*) OR (*Legionella bozemanii*) OR (*L. bozemanii*)) AND ((Growth) OR (Intracellular Growth) OR (Replication) OR (Reproduction) OR (dot/icm) OR (Genetic Knockout) OR (Genetic Knockdown) OR (Genes for growth) OR (Host interaction) OR (Disinfection) OR (*Acanthamoeba*) OR (*Acanthamoeba polyphaga*) OR (*Acanthamoeba castellanii*) or (*A. polyphaga*) OR (*A. castellanii*) OR (Premise Plumbing) OR (Biofilms) OR (Egress) OR (Exit) OR (Mediated death) OR (Mediated exit) OR (Apoptosis)”. Relevant citations were forward and reverse searched, and imported into a Zotero library.

### 2.2. Eligibility Criteria

The inclusion criteria were *Legionella* studies which looked at the bacteria’s behavior, protein function, genetic function, genetic expression, or genetic change while growing within or egressing from a host cell. Studies had to be peer-reviewed and written in English.

Studies were excluded if they were performed in mice, focused on lifecycle stages outside of growth or egress, focused on free *Legionella* or cells outside of a host cell, were abstracts from conference proceedings, were letters to the editor, or were not refereed.

### 2.3. Study Selection

Over 2000 papers were imported to a Zotero library to review for relevance. Zotero’s automation was used to remove duplicate items. Titles and abstracts were reviewed for relevance by two reviewers working independently, then sorted by lifecycle stages, protein, or gene function. Studies were then determined to be eligible for modeling purposes if they were (1) performed in triplicate (2) utilized microbial techniques which only accounted for growth of bacteria within, or undergoing egress from, the host cell, while excluding additional bacterial uptake in the host cell (3) accounted for time and (4) able to be combined with similar data from the literature for one working model. Thirty-two studies evaluating proteins and twenty studies evaluating gene function contributing to the growth or egress of *Legionella* in a host cell were included in this review. Six studies were included as viable options to model growth rate efficiency within the cell and four studies were included to model egress rate efficiency. To be included in the modeling studies, a change in growth or egression rates based on a genetic knockdown needed to be measured. Nine studies overlapped in these categories for a total of 53 included studies. The Preferred Reporting Items for Systematic Review and Meta-Analyses (PRISMA) guidelines were used for this review. The 2020 PRISMA checklist can be found in Supplemental Material S1.

## 3. Results

### 3.1. Lifecycle and Legionella Containing Vacuoles

*L. pneumophila* has multiple ways of invading the host cell, resulting in the bacteria becoming encased in the phagosomal membrane [10]. The phagosomal membrane will alter its thickness to resemble the thinner endoplasmic reticulum (ER) vesicle, and invade the rough endoplasmic reticulum (RER) about 6 h after infection [42]. The bacteria are then able to intercept vesicular traffic from the ER exit sites to create an organelle that permits intracellular replication, preventing the destruction of the host cell—the *Legionella* containing vacuole (LCV) [43]. In the first hour, the mitochondria cluster about the LCV. Approximately 4–8 h after phagocytosis, the host cell’s ribosomes appear on the cytoplasmic side of the vacuolar membrane while *L. pneumophila* multiplies in the vacuole. At the end of the eighth hour, nearly all of the LCVs are studded with ribosomes and the bacteria have a doubling time of about 2 h [44,45]. *L. pneumophila* will remain within the LCV until hundreds of bacteria fill the vacuole and the monocyte ruptures [46].

*L. pneumophila* utilizes the Dot/Icm Type IV Secretion System (T4SS) and the Type II Secretion System (TS2), in order to evade the phagosome-lysosome binding process, allowing the bacteria to replicate within the host cell [46,47,48]. These systems share several components, most likely having a common evolutionary origin [49,50,51]. Together, they export over 300 effector proteins. TS2 exports multiple effectors which contribute to the broad host range of *L. pneumophila* TS2, including protesase, RNase, lipase, phospholipase A, phospholipase C, lysophospholipase A, and tartrate-sensitive and tartrate-resistant acid phosphatase [52,53]. While the bacteria are replicating within the host cell, the LCV recruits host proteins in order to aid in LCV maintenance, including calnexin, Sec22b, BiP, SAR1, and Rab1, which are host factors involved in the endoplasmic reticulum (ER) recruitment process [54,55,56,57]. Some of these proteins are soluble in the cytosol and enter the LCV. They aide the LCV in avoiding intracellular degradation by establishing an ER-associated replicative compartment [54,58,59]. Individual effector proteins and host cell proteins will be discussed in further detail in Table 1.

LCVs that were expelled from host cells were found to have as many as 10^4^ *L. pneumophila* within a single vesicle [60]. More than 90% of vesicles containing viable *L. pneumophila* cells, expelled from *A. polyphaga* or *A. castellanii*, were of a respirable size, <5 µm [39]. It is theorized that one is more likely to become infected with *L. pneumophila* after inhaling a contaminated vesicle as opposed to free bacteria [61].

**Table 1:** Proteins associated with *L. pneumophila*’s ability to grow within and egress from a host cell.

Protein	Function	Target	Reference
AnkB	Translocated effector, allows proliferation of bacteria.	LCV	[47,62]
AnkG	Prevents apoptosis of host cell allowing for continued replication of *L. pneumophila* in mammalian hosts.	Host protein gCq1R(p32)	[63]
DsbA2	Catalyzes the disulfide bond formation required for the extracytoplasmic assembly of the T4SS system of *L. pneumophila.*	Dot/Icm T4SS	[64]
Dot/Icm Type 4 Secretion System (T4SS)	Translocates over 300 proteins into the host cells. Modulates host processes including phagosome-lysosome binding, promotion of ubiquitin conjugates, and suppression of dendritic cell formation.	Icm/Dot Translocated substrates (IDTS)	[65,66,67,68]
DrrA	Required for host cytotoxicity. Recruits and activates Rab1 on the plasma membrane-derived organelles.	Host vesicular transport	[56]
IcmSW	Mediates a conformational change facilitating T4SS recognition of the effector protein, thereby enhancing effector protein delivery	Translocation domain in the effector protein	[69]
IcmQ	Forms pores in lipid membranes by utilizing a chaperone/substrate relationship.		[70]
IcmR	Binds to the N-terminal of IcmQ inhibiting membrane insertion and pore formation.	IcmQ	[70]
LbtP	Sidephore transport protein which allows for growth in iron-limiting conditions. Prevents premature exit of macrophage due to low nutrients.		[26]
LbtU	Sidephore transport protein which imports iron-bound legiobactin. Alos for growth in iron-limiting conditions.	Legiobactin	[26]
LegC3	Inhibits SNARE and Rab GTPase dependent membrane fusion pathway		[71]
LegK1	Modulates macrophage defense and inflammatory response during infection of a host cell.	NF-kB	[72]
LegK2	Efficient recruitment of endoplasmic reticulum markers allowing for timely intracellular replication and ER uptake of the LCV.	T4SS	[73]
LtpD	Intracellular bacterial replication.	Phosphatidylinositol 3-phosphate	[74]
PieA	Avoids phagososome/lysosome binding. Allows for growth in the cell.	LCV	[75,76,77]
PI4P	Localization of effectors to LCV early during infection.		[48,78]
PmrA	Allows for intracellular growth in host cells.	*Dot/Icm* Type 4 secretion system	[79]
RalF	Exchange factor for the ARF family of GTPase. Required for the localization of ARF of LCV	ARF	[80]
Rap1	Allows for intracellular bacterial replication.		[81]
RpkA	Localizes endosomal membranes, specifically recruited to the phagosome.	LCV	[13]
RpoS	Stimulates intracellular replication and osmotic resistance. Growth phase stress resistance in protozoa. Maximum flagellin expression.	*fliA, flaA, mip*	[82,83,84]
SidC	Involved in recruiting host ER proteins to the surface of the LCV, allowing for intracellular bacterial replication.	LCV, PtdIns(4)P	[85]
SidF	Allows for more bacterial replication by making host cell resistant to apoptosis.	NIP3, Mcl-rambo	[86]
SidH	Important in early phase of infection. Inhibits cells death.		[87]
SetA	Allows for bacterial virulence in the post-exponential growth phase by preventing entry of the LCBB into the endocytic network.	LCV	[88]

### 3.2. Flagella

Similar to the various pathways *L. pneumophila* can use to infect host cells, the bacteria utilize flagellin differently within different hosts and during various stages of development. Flagellin and motility are required for the colonization of *A. castellanii* to activate the NLRC4 pathway, but have an adverse effect during bacterial replication in mammalian lungs [89]. *L. pneumophila* require flagellin to induce apoptosis of the host cell, but flagellin are not required for replication within the host cell [90]. The flagella initiates the caspase 9 and effector caspase 3, activating the pro-apoptotic protein Bax and inhibiting the anti-apoptotic protein X-linked inhibitor of apoptosis (XIAP) via the inhibition of the Akt pathway [90]. Macrophages and dendritic cells use flagellin to assess the virulence of bacteria [91]. Flagellin is responsible for activating the nuclear factor κB (NF-κB), p38 mitogen-activated protein kinases (MAPK), Jun N-terminal kinase (JNK), and transforming growth factor β-associated kinase 1 (TAK1), which induce interleukin-8 (IL-8) activation, the human immune response to *L. pneumophila* infection [92]. Nod-like receptors NOD1 and NOD2 are partially responsible for neutrophil recruitment and cytokine production in the mammalian lung [93,94,95]. Flagellin can be translocated by the Dot/Icm complex into the host cell cytosol, where macrophages and dendritic cells can use the protein to assess the virulence of the bacteria [91,96]. Although *L. pneumophila* cannot replicate within dendritic cells, it still utilizes the Dot/Icm complex to establish ER-derived LCV within the cell [97]. The interaction between the flagellin and the host cell Nod-like receptors, Ipaf and NLRC4, which both activate caspase 1 or TLR5, can induce the expression of pro-inflammatory cytokines [89,98,99]. NLRC4 is not localized to a distinct structure within the cell, allowing inflammasomes to gain access to different subsets of substrates. It also activates caspase 7, which promotes non-apoptotic functions such as LCV maturation and bacterial degradation [100].

### 3.3. Interferons

Type I interferons (IFN-α/β), which boost the immune system in response to an infection, are induced by *L. pneumophila* after the Dot/Icm complex translocates bacterial DNA into the cytosol of the host cell [101,102,103]. A downstream signaling adaptor in the stimulator of interferon genes (STING) pathway is required for type I IFN induction in response to an upstream sensor of the cytosolic DNA [104,105,106]. Type I IFNs directs the activation of both Stat 1 homodimers and IFN-stimulated gene factor 3 (ISGF-3) which are integral in the activation of the IFN-I autocrine loop [107]. Type II IFNs (IFN-γ) utilize the classical pathway to activate macrophages with only the Stat1 homodimer. Both Type I and Type II IFNs play an integral role in the innate immune response of the macrophage to intracellular microbes [107]. Even low doses of IFN are effective in preventing the replication of *L. pneumophila* in macrophage host cells [108]. IFN-activated macrophages inhibit the bacteria from proliferating mainly by reactive oxygen intermediate and reactive nitrogen intermediate independent mechanisms, and secondarily by nutritionally dependent mechanisms [109]. One key protein in suppressing the IFN response to *L. pneumophila*, SdhA, is discussed in Table 2 [110].

### 3.4. Mediated Cell Death

Naip5, a Nod-like receptor protein, initiates cell death through the activation of caspase 1, causing a pore formation and resulting in pyroptosis, caspase 1-mediated cell death [123,124,125,126,127]. Caspase 1 activation is mediated in response to a translocated Dot/Icm substrate and recombinant flagellin in the cytosol, and will not occur in IPAF mutant cells [100,123,128]. This process stimulates autophagy in macrophages, resulting in the redirected trafficking of the LCV to lysosomes [129,130].

Despite normal caspase 1 function, if the cytosolic protein NAIP5 is defective, the macrophage is permissive to the bacteria replicating [127,131]. All macrophages require a competent Dot/Icm complex in order for the cell to undergo apoptosis [132]. ASC, the apoptosis-associated speck-like protein, containing a caspase recruitment domain, is an adaptor protein not associated with pyroptosis, but rather aids in mediated-cell death via an independent inflammasome pathway [123]. ASC plays a role in the negative regulation of caspase 1-dependent host cell death [133]. ASC is required for the secretion of inflammatory cytokines IL-1β and IL-18 [123,128,134]. Cytokine processing occurs in a single, large, punctate structure in host cells, where ASC and caspase 1 are recruited [134]. Caspase 1 is required for efficient cytokine processing, as a mutant form of caspase 1 is unable to support cytokine cleavage [134].

Caspase 3 is essential for apoptosis in monocytes, macrophages, and alveolar epithelial cells. While the bacteria activate caspase 3 upon invasion, apoptotic death is not executed until late stages of infection, after the bacteria have completed replication [135]. The protein is associated with effective Dot/Icm mediated anti-apoptotic stimuli which cause the cell to resist the apoptotic inducer during bacterial replication [100]. The caspase proteins and non-apoptotic functions of executioner caspases are modulated, temporally and spatially, during infection, determining permissiveness to intracellular bacterial proliferation [100].

The host death of infected macrophages occurs in a biphasic model. The induction of apoptosis occurs during the early stages of infection and the independent and temporal induction of necrosis occurs during the late stages of intracellular replication [121]. In a similar manner as the bacteria kills the protozoan host, necrosis and cytolysis of macrophages by *L. pneumophila* is mediated by pore-forming activity or toxin [121,136]. The pore-forming activity is signaled after the bacteria have finished replicating in the host cell. Mutants which are incapable of pore-forming activity can replicate within the host cell but are unable to lyse the host cell and egress. They will eventually be released by the host, most likely by apoptotic death [121].

*L. pneumophila* utilizes multiple mechanisms to induce cell death, which can vary depending on the host cell. *L. pneumophila* uses type II and IV secretion systems to cleave large subunit ribosomal RNA resulting in decreased mitochondrial messenger RNAs in *Dictyostelium discoideum* (*D. discoideum*) [137]. However, even in other protozoan hosts, such as *A. castellanii*, *L. pneumophila* do not use this method. Contact-dependent cytotoxicity is required for the egress of *L. pneumophila* from the amoeba, while it is not needed for the bacteria to survive and thrive within the host cell [113]. In dendritic cells, mitochondria-regulated apoptosis occurs within 1 h [138]. This process is initiated by caspase 3 or BH3-only proteins [96].

### 3.5. Stress

Amoeba are known as a Trojan Horse for pathogenic microorganisms, serving as both reservoirs and vehicles for the bacteria in the environment [14]. Furthermore, amoeba can serve as a unicellular incubator, allowing the bacteria to adapt to life within a human macrophage, favoring pathogenesis [14]. Environmental stressors such as high temperatures, unfavorable pH, osmotic pressure, or presence of disinfectants can cause amoeba to encyst, a life-stage particularly protective to intracellular bacteria [61]. The double layered cyst is particularly resilient, surviving in temperatures from −20 °C to 42 °C and showing resistance against disinfectants, such as chlorine [139]. The amoeba will return to the trophozoic form when environmental conditions are more favorable. *L. pneumophila* have been observed in both trophozoites and cyst hosts [140]. Vesicles expelled from protozoan host cells have demonstrated resilience when exposed to biocides in cooling towers for up to 24 h, ultrasound, and vast temperature ranges (−70 °C to 35 °C) [39].

## 4. Discussion

### 4.1. L. pneumophila in Premise Plumbing Systems

Legionellosis is of particular concern in large premise plumbing systems, such as those found in hospitals or hotels, due to water stagnation, institutional hot water being kept under 50 °C, and disinfectant dissipating throughout the large systems [141,142,143,144]. The elderly and immunocompromised have higher incidence rates of LD, making hospitals and nursing care facilities of even greater concern [145,146,147]. Patients with ambulatory impediments may take longer to shower, leading to longer exposure times to *L. pneumophila* if it is present in the premise plumbing system [148]. Due to the seriousness of LD and the increasing incidences of legionellosis throughout the US, there have been many campaigns to eradicate the bacteria from premise plumbing systems, specifically in biofilms where the bacteria are significantly more difficult to treat than free living *L. pneumophila*. The endosymbiotic relationship *L. pneumophila* has with protozoa makes eradication of the bacteria within the biofilm of premise plumbing systems exceedingly difficult [149]. As *L. pneumophila* use the protozoa as a reservoir the bacteria are particularly resistant to typical disinfection measures [135]. *L. pneumophila*’s lifecycle and factors affecting the bacteria’s persistence and virulence have been well documented. However, a comprehensive literature review providing information necessary to model *L. pneumophila*’s growth within and egress from host cells within a biofilm was not available, necessitating this article.

### 4.2. Genetic Knockdowns

Genetic knockdowns or mutations would not only affect the individual bacterium, but extend to its progeny, affecting all future generations. Therefore, a knockdown or mutation that is nonlethal, but lowers the bacterium’s infectivity would then lower the infectivity of all of the bacterium’s descendants. Genetic knockdown information, which affects *L. pneumophila*’s ability to replicate within and egress from the host cell, was drawn from the literature. Data are only reported in this article if they were sufficient to use in a mechanistic model, requiring that: (a) they were reported in a peer-reviewed journal, (b) they had greater than three data points, (c) they were validated, (d) the genes effected are responsible for pathogenesis, and (e) they were comparable to the other data provided. Table 2 summarizes the functions of genes essential for *L. pneumophila* to grow within and egress from host cells. Variations in methodologies, timeframes, and units reported (i.e., log vs. percentage reduction) present a challenge in using data from the literature in one comprehensive model. In this article, the rates at which the modified bacteria had reduced capacity to grow within or egress from the host cell after the knockdown when compared to the wildtype are reported in the same units as the cited article in the “change in growth efficiency” or the “change in egress rate efficiency” columns in Table 3 and Table 4, respectively. The degradation of growth or egress rates are also expressed as percentages in Table 3 and Table 4, for the sake of uniformity in modeling. Percentages were chosen over log reductions as some genetic knockdowns cause only a minor change in growth or egress. For modeling purposes, it is recommended that future research report their results in percentage reductions. There is a great deal of redundancies within the gene functions in *L. pneumophila,* making the bacteria more robust to environmental stressors and adaptable to its environmental conditions. However, it is clear that specific genetic knockdowns, such as the ones described in Table 3 and Table 4, reduce the bacteria’s virulence in the biofilm and premise plumbing systems.

### 4.3. Contribution to the Literature

This study summarizes information from the literature, which is vital to model the growth within and egress from host cells, and how these mechanisms can be influenced by environmental stressors, such as disinfectants. In recent years the importance of replication within host cells for the virulence of *L. pneumophila* in human macrophages has become clear [35]. It is important to incorporate this knowledge into future predictive models so as to fully understand the infectivity of the bacteria to humans. This paper summarizes the information available in the literature to allow for said more substantial predictive models.

### 4.4. Limitation of This Systematic Review

This review serves to summarize the data available in the literature regarding how oxidative stress affects *L. pneumophila*’s lifecycle, specifically during its replication and egress phases. It looks at how oxidative stress affects genetic regulation in the bacteria and how those genes affect these lifecycle traits. However, there is still much work to be done in understanding the lifecycle of *L. pneumophila*, its interaction with the host cell, how environmental stressors change this relationship, and what genes are involved. Furthermore, this study did not focus on the antibiotic resistance of *L. pneumophila* in the environment or in humans. This will undoubtedly be a topic of high importance in the future and would be helpful to incorporate in monitoring for the bacteria and future risk analysis.

## 5. Future Directions

**Figure 1:**
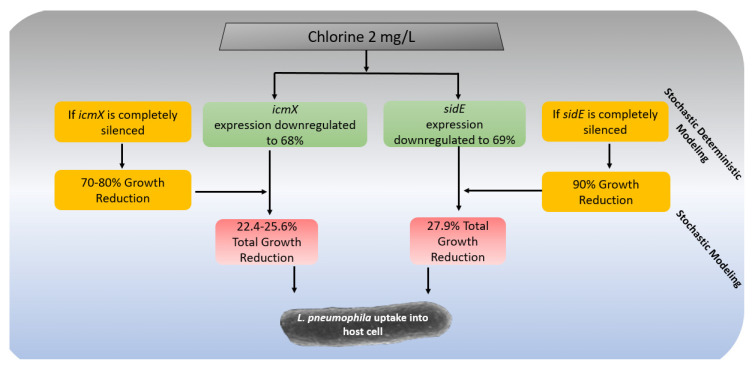
Modeling framework for intracellular growth of *L. pneumophila* in a host cell.

This review summarizes knowledge and data that can be used to build an intracellular growth model for *L. pneumophila* in the biofilm. It is clear that replication within a host cell is vital to incorporating infectivity and virulence while modeling the lifecycle of *L. pneumophila*. The genes that regulate protein secretion and ultimately the phylogenic characteristics of effective replication in and egress from the host cells are used to model the lifecycle of the bacteria. The stochastic method was used in modeling this data as a result of the uncertainty and variability of environmental stressors effects, as well as the lack of data conducive to modeling. Stochastic methods allow for systems and data uncertainty and variability to be accounted for and used in the model estimates. The modeling framework for the intracellular growth part of the predictive model is represented in Figure 1. The environmental quality and oxidative stress impact genetic knockdown which in turn affects phylogenetic outcomes, resulting in replication rate degradation. The modeling framework in Figure 1 allows for a mechanistic model of replication rates due to oxidative stress.

## Figures and Tables

**Table 2:** Genes associated with *L. pneumophila*’s ability to grow within and egress from a host cell.

Gene	Function	Target	Reference
*Dot/Icm* Complex	Avoids phagososome/lysosome binding. Allows for growth in the cell and genetic conjugation.	LCV	[75,76,77,111,112]
*ankB*	Intracellular replication in U937, *A. polyphaga*, and human monocyte-derived macrophages.		[62]
*ccm* locus	Growth, intracellular infection and virulence, especially in low iron environments	Cytochrome c	[25]
*dotA*	Intracellular replication within macrophages, required in the immediate stages of infection to prevent lysozyme fusion. Not required for growth within amoeba or nematodes.		[75,113,114,115,116,117]
*dotB*	Not required for intracellular growth in amoeba.		[113]
*dotO*	Infection of A549 alveolar epithelial cells.	Activates caspase 3, 8, 9, and 1. Released HMGB1.	[118]
*katA*	Bifunctional catalase-peroxidase. Keeps hydrogen peroxidase levels low in the cell allowing intracellular multiplication.	LAMP-1, recruits phagosomes	[113,119,120]
*katB*	Bifunctional catalase-peroxidase. Keeps hydrogen peroxidase levels low in the cell allowing for intracellular multiplication,	LAMP-1, recruits phagosomes	[113,119,120]
*pilD*	Intracellular growth in U937 and amoeba	Type II Secretion System	[53]
*rib*	Expression of pore-forming toxin/activity		[121]
*sidJ*	Growth in macrophage and amoeba		[115]
*sdjA*	Growth in protozoan, but not macrophages		[115]
*sdhA*	Prevention of cell death-Mutation has increased nuclear degradation, mitochondrial distribution, membrane permeability, and caspase activation	Type I IFN expression	[110,122]
*sdeC*	Efficient intracellular growth		[101]

**Table 3:** Effect of genetic knockdowns on the growth of *L. pneumophila* within a host cell.

Gene	Host Cell	Change in Growth Rate Efficiency ^1^	Degradation of Growth Rate ^2^	Process
*dotA*	U937	35–56%	35–56%	Phagosome–lysosome fusion occurs [77].
*dotA*	*A. Castellanni*	Incapable of replication	100%	Phagosome–lysosome fusion occurs [150]
*dsbA*	*A. castellanni*	½ log reduction	68%	Defective oxidative protein folding necessary for replication [151]
*icmQ*	U937	Incapable of replication	100%	Defective pore formation in the macrophage [152]
*icmR*	U937	1.5 log reduction	97%	Defective pore formation in the macrophage [152]
*icmS*	U937	1.75 log reduction	98%	Phagosome–lysosome fusion occurs [152]
*icmT*	U937	Incapable of replication	100%	Phagosome–lysosome fusion occurs [153]
*icmW*	U937	2 log reduction	99%	Phagosome–lysosome fusion occurs [153]
*katA*	*A. castellanni*	2 log reduction	99%	Susceptible to exogenous hydrogen peroxide [113]
*katB*	*A. castellanni*	2 log reduction	99%	Susceptible to exogenous hydrogen peroxide [113]
*lvgA*	U937	10-fold decrease	90%	Phagosome–lysosome fusion occurs [150]
*lvgA*	*A. castellanni*	10-fold decrease	90%	Phagosome–lysosome fusion occurs [150]

^1^ Expressed in units reported in the original literature. ^2^ Degradation of Growth Rate refers to the percentage of bacteria decrease within the host cell as compared to the wild-type.

**Table 4:** Effect of genetic knockdowns on the egress of *L. pneumophila* within a host cell.

Gene	Host Cell	Change in Egress Rate Efficiency ^1^	Degradation of Egress Rate ^2^	Process
*dotA*	U937	75–85%	75–85%	Defect in inserting pores in eukaryotic membranes [132]
*dotBCD*	U937	80%	80%	Defect in inserting pores in eukaryotic membranes [132]
*icmGCD*	U937	70–75%	70–75%	Defect in inserting pores in eukaryotic membranes [132]
*icmJB*	U937	65–80%	65–80%	Defect in inserting pores in eukaryotic membranes [132]
*icmT*	U937	90%	90%	Defect in pore-formation to egress from cell [153]
*legK2*	*A. castellanni*	1.5 log reduction	96.8%	Defect in ER recruitment on the LCV [73]
*rib*	U937	70–95%	70–95%	Defective in necrosis-mediated killing of the host cell [121]
rib	WI-26	85–98%	85–98%	Defective in necrosis-mediated killing of the host cell [121]

^1^ Expressed in units reported in the original literature. ^2^ Degradation of Egress Rate refers to the percentage of bacteria decrease egressing from the host cell as compared to the wild type.

## Data Availability

All data used in this study are included in the published article.

## Supplementary Materials

The following supporting information can be downloaded at: https://www.mdpi.com/article/10.3390/microorganisms10010141/s1, Supplementary Material S1: PRISMA 2020 Checklist.
